# Laparoscopic resection of a giant pelvic solitary fibrous tumor: a case report

**DOI:** 10.3389/fonc.2025.1578307

**Published:** 2025-10-02

**Authors:** Zhoulei Shi, Shuaijiang Yan, Chunhong Yu, Liuxiong Guo, Panying Zhang, Jiarui Cui, Shoubin Li

**Affiliations:** ^1^ North China University of Science and Technology, Tangshan, China; ^2^ Department of Urology, Hebei General Hospital, Shijiazhuang, Hebei, China; ^3^ Department of Health Examination Center, Hebei General Hospital, Shijiazhuang, Hebei, China

**Keywords:** giant pelvic solitary fibrous tumor, laparoscopic surgery, diagnosis, treatment, prognosis

## Abstract

**Objective:**

To improve the understanding, diagnosis, and treatment of giant pelvic solitary fibrous tumor (SFT).

**Methods:**

We report a case of a giant pelvic SFT treated in our hospital. The clinical features, pathological findings, and treatment approach are described, and the current status of diagnosis, treatment, and prognosis is discussed with reference to the literature.

**Results:**

A 69-year-old male patient was admitted due to progressive dysuria for over 6 months and was diagnosed with a pelvic SFT. The patient underwent laparoscopic resection of the pelvic SFT. Postoperative pathological examination revealed a spindle cell tumor, consistent with a solitary fibrous tumor based on immunohistochemical staining. The patient recovered well postoperatively and remains under close follow-up.

**Conclusion:**

Pelvic SFT are clinically rare and exhibit unique pathological characteristics. Compared to traditional open surgery, this case demonstrates that laparoscopic surgery may be a superior option for large pelvic masses.

## 1 Introduction

Solitary fibrous tumor (SFT) is a rare mesenchymal tumor, accounting for less than 2% of all soft tissue tumors (1). SFT occurring in the pelvis are even rarer. Here, we report a case of a giant pelvic SFT successfully resected laparoscopically and discuss its clinicopathological features and treatment outcomes.

## 2 Case presentation

**Figure 1:**
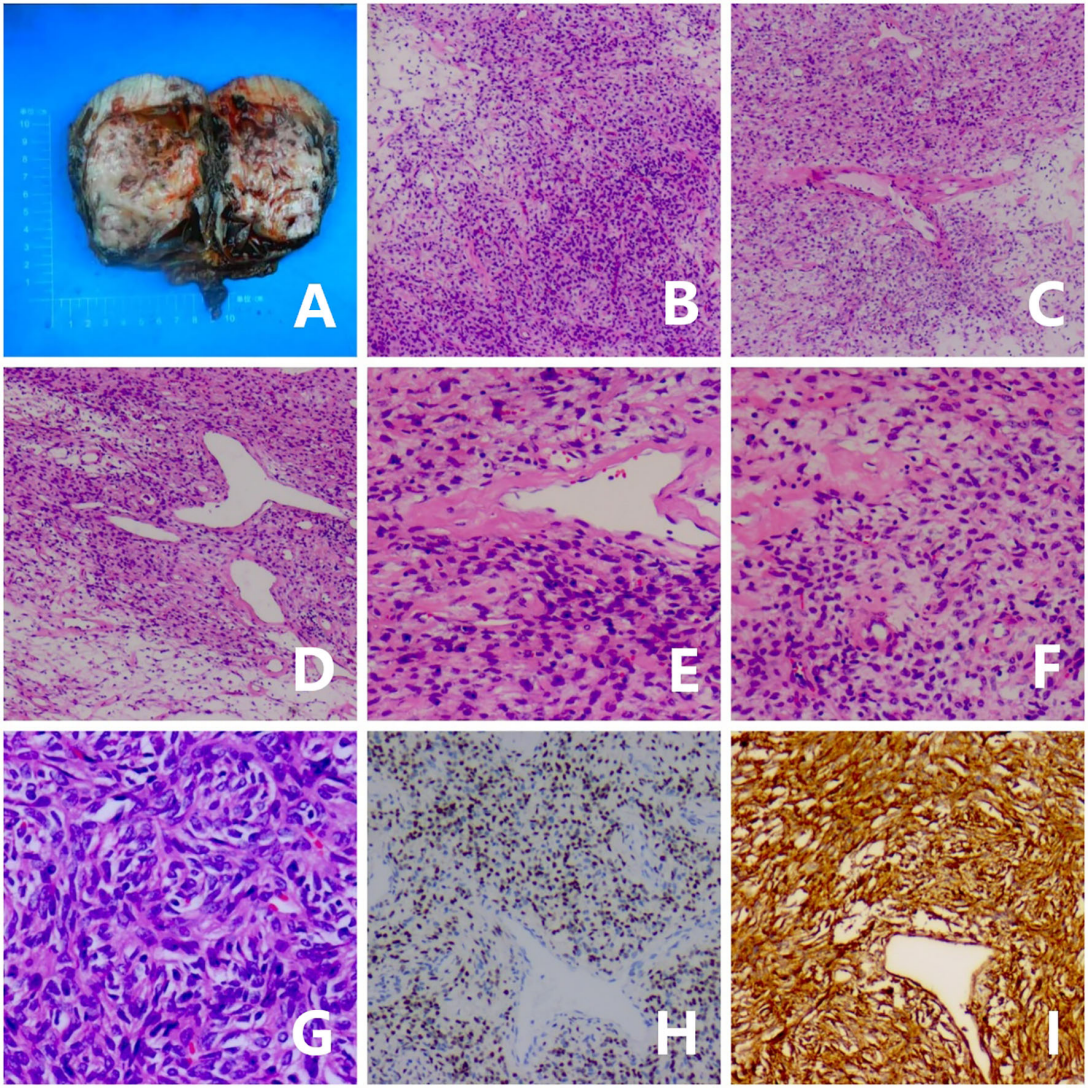
Gross, microscopic, and immunohistochemical features of the tumor. **(A)** Gross appearance of the tumor. **(B)** The tumor shows hypercellular and hypocellular areas (×40 magnification). **(C, D)** The tumor is composed of spindle-shaped tumor cells with varying amounts of collagenous stroma and staghorn-shaped vessels (×40 magnification). **(E)** Spindle-shaped tumor cells and staghorn-shaped vessels (×100 magnification). **(F)** The spindle tumor cells exhibit mild cytologic atypia with visible collagen bundles (×100 magnification). **(G)** Tumor cells have scant cytoplasm, oval nuclei, and inconspicuous nucleoli (×200 magnification). **(H)** STAT6 shows strong and diffuse nuclear expression in tumor cells (×100 magnification). **(I)** CD34 is positively expressed in tumor cells (×100 magnification).

**Figure 2:**
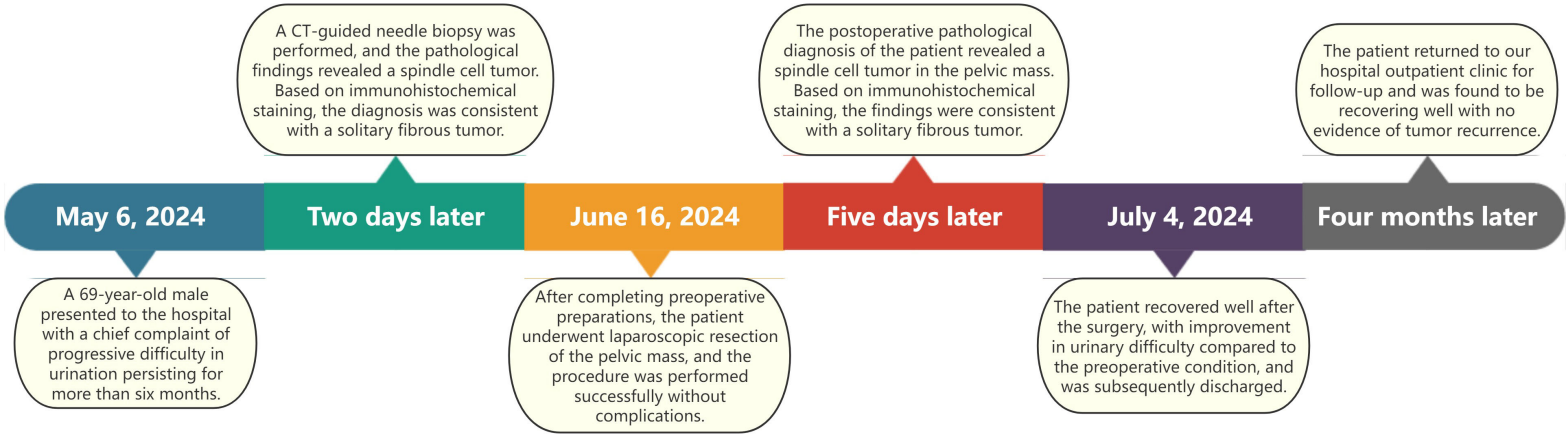
Treatment timeline of this case.

A 69-year-old male patient presented with a 6-month history of progressive dysuria, accompanied by urinary frequency but no urgency, dysuria, hematuria, fever, nausea, vomiting, or weight loss. Pelvic CT scan with 3D reconstruction showed a mixed-density mass measuring approximately 106×80× 94 mm was observed in the left pelvis. The mass had no clear boundaries from the left seminal vesicle and exerted compression on the bladder, prostate, and rectum. A CT-guided biopsy was performed, and pathological examination revealed a spindle cell tumor, consistent with a solitary fibrous tumor based on immunohistochemical staining. Immunohistochemical results were as follows (**Figure 1**): STAT6 (nuclear +), CD34 (+), CKpan (-), Vimentin (+), S100 (scattered +), Desmin (-), SMA (scattered +), EMA (-),and Ki-67 (approximately 2% +). After preoperative preparation, the patient underwent laparoscopic resection of the pelvic mass via a transabdominal approach. A 1 cm incision was made above the umbilicus for the observation port, and a TROCAR was inserted for the 3D laparoscope. No intestinal injury was observed. Additional TROCARs were placed 3 cm below the umbilicus on both sides, lateral to the rectus abdominis and 3 cm medial to the iliac crest. The pelvic mass was found to be significantly adherent to the bladder and prostate and was carefully dissected under 3D laparoscopy and completely removed. Postoperative pathological diagnosis confirmed a spindle cell tumor, consistent with a solitary fibrous tumor based on immunohistochemical staining, with 3 mitoses per 10 high-power fields (HPF), no tumor necrosis, and a risk score of 4 (moderate risk). The tumor did not involve the seminal vesicle or vas deferens, and the margins were tumor-free. Immunohistochemical results were consistent with the preoperative findings. The patient recovered well postoperatively, with significant improvement in urinary symptoms. At the 4-month follow-up, the patient showed no signs of recurrence.The treatment timeline for this patient is summarized in **Figure 2**.

## 3 Discussion and conclusion

SFT was first described by Klemperer et al. (2) in 1931 as a pleural lesion. Since then, cases have been reported in the lungs, mediastinum, pericardium, and other locations. In recent years, extrapleural SFTs have been increasingly reported. Current evidence indicates that the *NAB2-STAT6* gene fusion occurs with high frequency in SFT. Overexpression of the *NAB2-STAT6* fusion gene has been shown to promote proliferation in cultured cells and activate the expression of Early Growth Response (EGR)-related target genes, thus playing a critical role in tumorigenesis (3).It is a mesenchymal tumor that can occur in any part of the body. SFT can occur at any age but is most common in adults aged 20–70 years (4).

### 3.1 Clinical features of pelvic SFT

In the early stages, patients with SFT often lack specific clinical manifestations. Most patients present with local symptoms due to compression of adjacent structures by the enlarging mass or are incidentally found to have a large mass during physical examination. In this case, the patient’s symptoms were due to dysuria caused by bladder compression, consistent with the clinical features of SFT. Preoperative imaging cannot definitively diagnose SFT, and diagnosis relies on pathological and immunohistochemical findings.

### 3.2 Pathological examination of pelvic SFT

Microscopically, SFT cells are typically oval or short spindle-shaped without atypia, with indistinct cell boundaries. The stroma may show varying degrees of myxoid change, with vacuolated nuclei and scattered chromatin. Large branching or “staghorn” thin-walled vessels are often seen, and medium-sized vessels with perivascular hyalinization are common. Malignant SFT is characterized by infiltrative margins, pleomorphism, cellular proliferation, necrosis, and more than 4 mitoses per 10 HPF (5). Immunohistochemically, the most valuable diagnostic marker for SFT is STAT6, for which strong nuclear positivity demonstrates high sensitivity (95.8%) and specificity (88.3%) (6), establishing it as the current diagnostic gold standard. Additionally, SFT often shows expression of ancillary markers such as CD34, Bcl-2, and CD99, which serve as supportive diagnostic indicators.

### 3.3 Treatment of pelvic SFT

The primary treatment for SFT is surgical resection. Preoperative evaluation should thoroughly assess the tumor’s relationship with surrounding tissues and organs, and a comprehensive surgical plan should be formulated. It is recommended that the resection margin be at least 2 cm from tumor, as complete resection is essential to preventing recurrence (7). For tumors with malignant potential that cannot be completely resected, postoperative radiotherapy or targeted therapy may be considered.

### 3.4 Prognosis of pelvic SFT

Most patients have a good prognosis if the tumor is completely resected. However, due to the tumor’s tendency to recur, especially in cases with confirmed malignant histopathology, close postoperative follow-up is essential.

Currently, the majority of the literature reports that the surgical treatment for SFT is primarily performed via open laparotomy. Laparoscopic surgery offers several advantages, such as clear and magnified visualization, allowing for more precise dissection and minimizing collateral damage; smaller incisions for better cosmesis; and faster postoperative recovery. For large masses in the narrow pelvic space, laparoscopic surgery provides better access to deep pelvic tissues. The successful laparoscopic resection in this case suggests that laparoscopic surgery may be a superior option for large pelvic masses.

## Data Availability

The original contributions presented in the study are included in the article/Supplementary Material. Further inquiries can be directed to the corresponding author.
